# Comparison of Vinorelbine-Cisplatin with Gemcitabine-Cisplatin in Patients with Advanced Non-Small Cell Lung Cancer

**DOI:** 10.4137/ccrpm.s578

**Published:** 2008-04-18

**Authors:** Sevket Ozkaya, Serhat Findik, Oguz Uzun, Atilla Guven Atici, Levent Erkan

**Affiliations:** 1 Specialist; 2 Associate Professor; 3 Assistant Professor; 4 Professor, Department of Pulmonary Medicine Faculty of Medicine Ondokuz Mayis University SAMSUN/TURKEY

**Keywords:** non-small cell lung cancer, median survival, cisplatin, gemcitabine, vinorelbine, toxicity

## Abstract

**Purpose:**

The objective of this trial was to compare cisplatin-plus-vinorelbine regimen with cisplatin-plus-gemcitabine regimen in patients with stage IIIB-IV non-small cell lung cancer (NSCLC).

**Patients and Methods:**

Chemonaive patients with stage IIIB-IV NSCLC received either vinoelbine 30 mg/m^2^ (days 1 and 8) plus cisplatin 80 mg/m^2^ (day 1) every 21 days (VC arm) or gemcitabine 1250 mg/m^2^ (days 1 and 8) plus cisplatin 80 mg/m^2^ (day 1) every 21 days (GC arm).

**Results:**

One hundred thirtyfour patients (67 VC and 67 GC) were included to the study. Overall response rates for the VC arm (31.2%) were not significantly different from that of the GC arm (34.3%). There were no differences in overall survival and one-year survival rates. Median survival and one-year survival rates for the VC and GC groups were 10.6 and 11.5 months, 45% and 46.8%, respectively. Grade 3–4 thrombocytopenia was significantly higher on the GC arm (VC 1.4% v GC 8.9%, p < 0.05), as was febrile neutropenia on the VC arm (VC 8.9% v GC 1.4%, p < 0.05).

**Conclusion:**

VC and GC demonstrated similar efficacy but there were differences in toxicity profiles.

## Introduction

Lung cancer is the leading cause of cancer deaths in both men and women. Appoximately one third of all cancer related deaths are due to lung cancer, which accounts for more deaths each year than breast, prostate, and colon cancer combined (Jemal et al. 2002).

Seventy-five percent of patients with lung cancer present in inoperable, locally advanced (stage IIIB) or metastatic disease (stage IV) and are therefore candidates for some forms of systemic chemotherapy. Patients with advanced non-small cell lung cancer (NSCLC) have a poor outcome, with a survival time of approximately 4 to 6 months and 1-year survival rate of 10%–15% (Ginsberg et al. 1997).

Chemotherapy for advanced NSCLC was often considered ineffective or excessively toxic. However, meta-analyses have demonstrated that, as compared with supportive care, chemotherapy resulted in a small improvement in survival in patients with advanced NSCLC (Marino et al. 1994; Non-small Cell Lung Cancer Collaborative Group 1995; Grilli et al. 1993). In addition, randomized studies comparing chemotherapy with the best supportive care have shown that chemotherapy reduces symptoms and improves the quality of life. (Cullen et al.1999).

Over past decade, third generation agents such as vinorelbine, taxanes, gemcitabine and irinotecan have been introduced to the treatment of NSCLC. The combination of one or more of these agents with a platinum compound has resulted in high response rates and prolonged overall survival. However, there have been few comparisons of these newer chemotherapy regimens, which are now used frequently, with each other.

Therefore, we decided to investigate the activity and toxicity of two commonly used regimen s cisplatin and vinorelbine (VC) or cisplatin and gemcitabine (GC) in patients with stage IIIB or IVin this trial. The primary objective of this study was to compare overall survival.

## Patients and Methods

We identified consecutive patients with advanced stage NSCLC in our clinic. Data including demographic values, medical histories, symptoms and signs, laboratory examination results, and radiologic and scintigraphic documents were taken from files of the patients and archives of the department, respectively. Performance status was classified in accordance with the criteria of European Cooperative Oncology Group (ECOG). Staging was conducted in accordance with WHO and TNM by evaluation of the imaging methods such as chest x-ray, thoracic computed tomography, abdominal computed tomography, abdominal ultrasonography (USG), cranial computed tomography and bone scintigraphy.

### Eligibility criteria

The criteria for eligibility included confirmed disease, measurable or nonmeasurable; an age of at least 18 years; adequate hematologic function (as indicated by a white-cell count of at least 4.000 per cubic millimeter and a platelet count of at least 100.000 per cubic millimeter), hepatic function (as indicated by a billirubin level that did not exceed 1.5 mg per decilitre, and AST and ALT levels being less than three times of the normal values), and renal function (as indicated by a creatinine level that did not exceed 1.5 mg per deciliter); and ECOG performance status of ≤2.

### Exclusion criteria

Criteria as to the exclusion from the study were as follows: insufficient hematological, renal and hepatic functions; instable brain metastasis; history of prior chemotherapy and/or radiotherapy; presence of uncontrolled infections; presence of an additional malignancy; presence of a systemic disease contradicting administration of chemotherapy; pregnancy; performance status of >3; unfitness for follow-up due to psychological, familial, sociological and geographical reasons.

### Treatment regimens

The main treatment was chemotherapy for patients with stage IV disease and sequential chemoradiotherapy for patients with stage IIIB disease.

### Chemotherapy regimens

#### Cisplatin-plus-vinorelbine (VC) regimen

Vinorelbine, at a dose of 30 mg per square meter, was administered on days 1, and 8, and cisplatin, at a dose of 80 mg per square meter on day 1 of a three-week cycle. The cycle was repeated every three weeks.

#### Cisplatin-plus-gemcitabine (GC) regimen

Gemcitabine, at a dose of 1250 mg per square meter, was administered on days 1, and 8, and cisplatin, at a dose of 80 mg per square meter on day 1 of a three-week cycle. The cycle was repeated every 21 days.

At least two cycles were given to the patients were considered assessable for response. Patients who responded to the treatment and did not show signs of toxicity or progression were given four to six cycles. Dosage was adjusted according to hematological, neurological, renal and hepatic functions. Dosage was decreased by 25% for patients who were classified as Grade III or Grade IV in accordance with WHO toxicity criteria.

### Radiotherapy

Curative radiotherapy was administered for all patients with stage IIIB disease who had responded to chemotherapy after three cycles of chemotherapy regimens. And remaining one to three cycles were given after three weeks to one-month after administration of radiotherapy. The doses of the thoracic radiotherapy was 60 Gy.

### Outcome criteria

The aim of this nonrandomized comparison of retrospective study to evaluate the activity (response, overall survival) and toxicity of cisplatin-plus-vinorelbine and cisplatin-plus-gemcitabine regimens in patients with stage IIIB–IV non-small cell lung cancer. Standard ECOG response criteria were used. The response evaluated by thorax CT scan at after three cycles of chemotherapy and end of the treatment. Briefly, a complete response was defined as the absence of disease at all known sites for at least four weeks. A partial response was defined as a 50 percent reduction in the sum of the perpendicular diameters of all measurable lesions, lasting at least four weeks. Progressive disease was defined as either a 25 percent increase in the area of any one lesion over the prior measurement or the development of one or more new lesions. Survival was calculated from the date of diagnosis to the date of death or the date when the patient last known to be alive.

All patients gave written informed consent.

### Statistical analysis

Data was evaluated by using SPSS 10.0 programme (SPSS Inc., Chicago, IL, U.S.A). Survival of the patients was calculated from the date of diagnosis to the date of death. Response rates were calculated for patients with complete or partial responses. Median age, smoking habits, performance status, response rates and toxicity results of the groups were compared by using “Mann-Whitney U” and “Pearson chi-square” tests. Median survival and one-year survival rates of the groups were calculated by Kaplan–Meier method (Kaplan et al. 1958). Median survival rates were compared by using log-rank test. In all tests, p value was considered to be significant when it was 0.05 or less.

## Results

A total of 134 patients (67 patients for the VC group and 67 patients for the GC group) were enrolled in the study between January 2001 and September 2004.

The baseline characteristics of the patients in the two groups were similar (Table 1). The median age was 60.2 years. Almost 97 percent of the patients were men. Only 5.2 percent of the patients were non-smoker. 89.4 percent of patients had a performance-status score of 1, and 2. The median age was 58.9 years in VC and 61.6 years in GC group. About 71.6 percent of the patients had squamous carcinoma, and 64.9 percent had stage IIIB disease. There was no statistical difference between the groups with respect to baseline clinical characteristics of the patients. The median number of cycles was 3.5 for patients in VC group and 3.8 for those in GC group.

**Figure 1:**
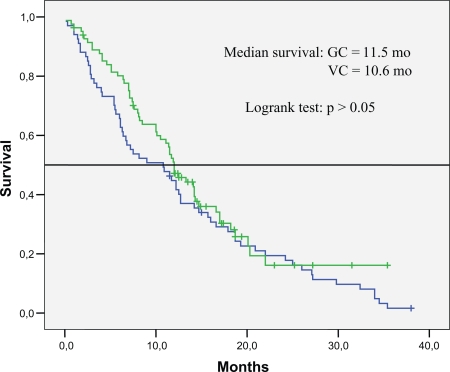


Table 2 presents outcome results of the treatment groups. The overall response rate for the 134 eligible patients was 32.7 percent. There were no significant differences in the response rate or survival among the groups. The response rates were 31.2 percent for patients in VC group and 34.3 percent for patients in GC group. The median survival was 10.6 months among the patients who received VC, 11.5 months among those who received GC. The one-year survival rate was 45 percent for patients who received VC and 46.8 percent for patients who received GC (Table 3 and Fig. 1).

The median survival was 11.2 months among the patients with stage III B disease treated with VC and 11.7 months among the patients with stage III B disease who received GC. For patients with stage IV disease the median survival was 9.7 months, and 11.0 months, respectively.

All patients were evaluated for toxicity. Table III shows toxic effects of the treatment groups. No treatment-related death was observed and no patient was withdrawn from the study due to toxicity. The major hematological toxicities encountered in this study were neutropenia, febrile neutropenia, thrombocytopenia and anemia. Percentages of both grade 3–4 anemia and grade 3–4 neutropenia were similar to each other in two groups. Febrile neutropenia was seen in 6 patients (8.9%) who received VC and in only 1 patient (1.4%) who received GC and this difference was statistically significant (Pearson chi-square test, p = 0.02). Grade 3–4 thrombocytopenia occurred in 1 patient (1.2%) who received VC and in 6 patients (8.9%) who received GC, the difference was statistically significant (Pearson chi-square test, p < 0.05). No serious hemorrhagic events were noted on either regimens.

Non-hematological toxicity was minimal. Grade 3–4 nausea and vomiting were observed only in 3 patients (4.4%) in VC arm and in 2 patients (2.9%) in GC arm. And the difference was not statistically significant.

## Discussion

Platinum-based chemotherapy regimen for advanced stage non-small-cell lung cancer results in a small but statistically significant improvement in survival, as compared with best supportive care (Marino et al. 1994). Chemotherapy in non-small cell lung cancer: a metaanalysis using updated data on individual patients from 52 randomised clinical trials (Non-small Cell Lung Cancer Collaborative Group, 1995; Grilli et al. 1993).

Although older chemotherapy regimens (e.g. mitomycin, ifosfamide, and cisplatin) resulted in survival rates of 10 to 15 percent at one year, second-generation regimens (e.g. cisplatin and etoposide) have resulted in survival rates of 20 to 25 percent at one year. The combination of the platinum analogues with the third generation drugs produce overall response rates of 30%–40%, median survival of 8 to 10 months, and 1-year survival rate of 30%–40% (Blackhall et al. 2004).

The evaluation of 10 randomized studies and 4 meta-analyses showed that combined chemotherapies and platinum-based chemotherapies provided a 10% increase in 1-year survival rate with a median survival of up to 25 weeks. Our study showed that third-generation regimens result in survival rates of 45 to 46.8 percent at one year. (Cullen et al. 1999; Cartei et al. 1993; Cellerino et al. 1991; Cormier et al. 1982; Ganz et al.1989; The Elderly Lung Cancer Vinorelbine Italian Study Group, 1999; Kaasa et al. 1991; Quoix et al. 1991; Rapp et al. 1988; Woods et al. 1990; Marino et al. 1994; Non-small Cell Lung Cancer Collaborative Group 1995; Grilli et al. 1993; Souquet et al. 1993).

We sought to determine whether there was a difference in between two newer third-generation chemotherapy regimens, with respect to survival. There was no statistically significant difference in survival between two regimens though patients in GC arm (11.5 months) had one month longer median survival than those in VC arm (10.6 months). One-year survival rates were 45% and 46.8% for VC arm and GC arm, respectively (p > 0.05). The overall response rate was 32.7 percent; the response rates were 31.2 percent for patients in VC group and 34.3 percent for patients in GC group. And there were no significant differences in the response rates between two groups.

In a multicentre phase III study, 272 patients were randomised to receive either vinorelbine 25 mg/m^2^ on days 1 and 8 plus cisplatin 75 mg/m^2^ on day 1 (regimen A) or gemcitabine 1200 mg/m^2^ on days 1 and 8 plus cisplatin 75 mg/m^2^ on day 1 (regimen B) (Martoni et al. 2005). Both treatments were recycled every 21 days. The following response rates were observed in regimens A and B, respectively: complete response was 0.7% and 3.7%, partial response was 31.9% and 22.2% (p = 0.321). Median overall survival was 11 months for both groups. Grade III–IV neutropenia occurred in 30.7% and 17.7% of the patients in arms A and B, respectively (p = 0.017); thrombocytopenia occurred in 0% and 9.3% (p = 0.004), respectively. Anemia rates were similar in both groups. A comparison of these data with ours showed that we had similar response rates with survival values despite the higher rate of complete response in our study. However, the toxicity values were lower in our study except for thrombocytopenia in vinorelbine arm. Consequently, both studies showed no statistically significant superiority for VC or GC over each other.

A large, multicenter, randomised trial conducted by the Italian Cancer Project compared gemcitabine 1250 mg/m^2^ days 1 and 8 plus cisplatin 75 mg/m^2^ day 2 every 21 days (gemcitabine arm), or paclitaxel 225 mg/m^2^ then carboplatin (area under the concentration-time curve of 6 mg/mLmin), both on day 1 every 21 days (PCb arm), or vinorelbine 25 mg/m^2^/wk for 12 weeks then every other week plus cisplatin 100 mg/m^2^ day 1 every 28 days (vinorelbine arm) (Scagliotti et al. 2002). Overall response rates for the gemcitabine (30%) and PCb (32%) arms were not significantly different from that of the vinorelbine arm (30%). There were no differences in overall survival, time to disease progression, or time to treatment failure. Median survival for the gemcitabine, PCb, and vinorelbine groups was 9.8, 9.9, and 9.5 months, respectively. Neutropenia (43%) was significantly higher on the vinorelbine arm and thrombocytopenia (16%) on the gemcitabine arm. Their conclusion was that efficacy end points were not significantly different between arms, although toxicities showed differences. A comparison of these values to ours revealed that efficacy and toxicity (especially neutropenia) values of our study were better that may be due to (1) higher percentage of stage IIIB patients versus stage IV patients (58% vs 42% in vinorelbine arm, 71.6% vs 28.3% in gemcitabine arm) in our study than those of Italian Cancer Project (19% vs 81% in both gemcitabine and vinorelbine arms) and (2) weekly administration of vinorelbine in Italian Cancer Project rather than on days 1 and 8 every 21 days.

Since there are a few studies comparing VC with GC in advanced stage non-small-cell lung cancer, we also included results of studies that compared VC or GC with other regimens (Le Chevalier et al. 1994; Wozniak et al. 1998; Schiller et al. 2002; Abratt et al. 1997; Anton et al. 1998; Comella et al. 2000; Cappuzzo et al. 2000; Huisman et al. 2001; Kelly et al. 2001; Fossella et al. 2003). In these studies, a response rate of 22%–54%, a median survival of 8.1–14.3 months and a one-year-survival rate of 33%–58% were reported for GC, response rate of 26%–57%; median survival of 7.1–11 months and one-year-survival rate of 28%–41% were reported for VC.

Apparently, the findings of our study are closer to the higher values in the literature. This may be due to the fact that in our study the number of stage IIIB patients is higher than that of the stage IV patients and that the number of patients who have two or more metastases is low. Another reason may be the regional differences but we were not able to compare our study with other studies due to lack of studies involving large series of patients in our country. The contrasts of results between our study and these studies may be related to differences at doses and schemes of cisplatin, vinorelbine and gemcitabine.

Toxicity results of our study showed that grade 3–4 thrombocytopenia occurred in 1.4% and 8.9% of the patients in VC and GC, respectively (p < 0.05); febrile neutropenia occurred in 8.9% and 1.2% (p < 0.05), respectively. Grade 3–4 anemia, grade 3–4 neutropenia and grade 3–4 nauseavomiting rates were similar in both groups. There is a wide variety in toxicity rates of previous studies that report grade 3–4 anemia as 7%–24% and 20%–30% of the patients in VC and GC, respectively; grade 3–4 neutropenia as 5.4–38.5% and 13.8%–81%; grade 3–4 thrombocytopenia rates as 2.5%–20% and 2.5%–6%; and grade 3–4 nausea-vomiting as 0%–58% and 3.2%–39%, respectively (Comella et al. 2000; Kelly et al. 2001; Crino et al. 1999; Cardenal et al. 1999). Our toxicity results were consistent with those of previous studies.

In conclusion, no significant difference in survival was observed between VC and GC regimens, although patients in the latter regimen had one month longer survival. On the basis of toxicity results, grade 3–4 thrombocytopenia and febrile neutropenia were significantly higher in GC and VC, respectively.

## Figures and Tables

**Table 1.** Baseline characteristics of the patients.

**Characteristic**	**Cisplatin and vinorelbine (N = 67)**	**Cisplatin and gemcitabine (N = 67)**
Age (yr)		
Median	58.9	61.6
Range	34–79	44–78
Sex (% of patients)		
Female	4.5	1.49
Male	95.5	98.5
Smoking status (% of patients)		
Nonsmoker	7.5	2.9
<10 pack/year	0	0
10–20 pack/year	7.5	8.9
21–30 pack/year	20.9	25.3
>30 pack/year	64.1	62.6
Performance status (% of patients)		
0	5.9	5.9
1	47.7	47.7
2	46.2	46.2
Disease stage (% of patients)		
IIIB	58.2	71.6
IV	41.8	28.3
Histological type (% of patients)		
Squamous cell	64.1	79.1
Adenocarcinoma	19.5	13.4
Undifferentiated NSCLC	16.4	7.4
Metastasis sites (no. of patients)		
1	32	20
2	7	6
≥3	3	2
Brain metastasis (no. of patients)	12	7

* Mann-Whitney U test,

** Pearson chi-square test.

**Abbreviations:** CV: Cisplatin-Vinorelbine; CG: Cisplatin-Gemcitabine; NSCLC: Non-small cell lung carcinoma.

**Table 2.** Outcomes of treatment groups.

**Variable**	**Cisplatin and vinorelbine (N = 67)**	**Cisplatin and gemcitabine (N = 67)**	**P**
Response-%			
Complete response	7.4	5.9	
Partial response	23.8	28.4	
Stable disease	49.2	35.8	
Progressive disease	19.4	29.8	
Overall response rate-%	31.2	34.3	0.71*
Survival			
Median (95% CI)-mo	10.6 (8.7–12.5)	11.5 (10.0–13.0)	0.45**
One-year survival-%	45	46.8	

* Pearson chi-square test.

** Log-rank test.

**Abbreviations:** CV: Cisplatin + Vinorelbine; CG: Cisplatin + Gemcitabine.

**Table 3.** Toxic effects.

**Type of toxic effect**	**Cisplatin and vinorelbine (N = 67)**	**Cisplatin and gemcitabine (N = 67)**
	% of patients	
Anemia		
Grade 1–2	46.2	41.7
Grade 3–4	8.9	7.4
Neutropenia		
Grade 1–2	44.7	35.8
Grade 3–4	1.4	8.9
Febrile neutropenia	8.9*	1.4
Thrombocytopenia		
Grade 1–2	5.9	11.9
Grade 3–4	1.4	8.9*
Nausea and vomiting		
Grade 1–2	65.6	58.2
Grade 3–4	4.4	2.9

Pearson chi-square test.

* p < 0.05.
